# The Fundamental Neutron Physics Facilities at NIST

**DOI:** 10.6028/jres.110.013

**Published:** 2005-06-01

**Authors:** J. S. Nico, M. Arif, M. S. Dewey, T. R. Gentile, D. M. Gilliam, P. R. Huffman, D. L. Jacobson, A. K. Thompson

**Affiliations:** National Institute of Standards and Technology, Gaithersburg, MD 20899-8461; National Institute of Standards and Technology, Gaithersburg, MD 20899-8461; Physics Department, North Carolina State University, Raleigh, NC 27695-8202; National Institute of Standards and Technology, Gaithersburg, MD 20899-8461

**Keywords:** cold neutrons, electroweak interactions, neutron interferometer, polarized neutrons, spin filters, ultracold neutrons

## Abstract

The program in fundamental neutron physics at the National Institute of Standards and Technology (NIST) began nearly two decades ago. The Neutron Interactions and Dosimetry Group currently maintains four neutron beam lines dedicated to studies of fundamental neutron interactions. The neutrons are provided by the NIST Center for Neutron Research, a national user facility for studies that include condensed matter physics, materials science, nuclear chemistry, and biological science. The beam lines for fundamental physics experiments include a high-intensity polychromatic beam, a 0.496 nm monochromatic beam, a 0.89 nm monochromatic beam, and a neutron interferometer and optics facility. This paper discusses some of the parameters of the beam lines along with brief presentations of some of the experiments performed at the facilities.

## 1. Introduction

Since the early 1990s, the National Institute of Standards and Technology (NIST) Center for Neutron Research (NCNR) in Gaithersburg, MD has provided neutron beam lines to an increasing number of fundamental experiments utilizing cold and ultracold neutrons. The experiments address important issues in nuclear, particle, and astrophysics such as unitarity of the Cabibbo-Kobayashi-Maskawa matrix, time-reversal invariance, the weak interaction between nucleons, nuclear three-body forces, and the abundance of ^4^He in the universe. Four beams are currently in use for fundamental physics experiments. These include three monochromatic neutron beams and a high-intensity polychromatic beam. In addition, a neutron imaging station and thermal-neutron column are available for users. The thermal column provides a large-area and uniform beam of thermal neutrons for dosimetry and neutron standards work.

The NCNR operates a 20 MW research reactor that provides a source of fission neutrons that have been moderated to thermal energies by the D_2_O primary reactor coolant. The peak core fluence rate is 4 × 10^14^ cm^−2^ s^−1^. Cold neutrons are produced by a neutron moderator situated adjacent to the reactor core. The cold source was recently upgraded and consists of an ellipsoidal shell of 5 L of liquid hydrogen maintained at a temperature of 20 K [1]; its brightness is approximately 1.5 × 10^13^ cm^−2^ s^−1^sr^−1^nm^−1^. Scattering of neutrons in the cold source cools the neutrons to approximately 40 K (they do not reach thermal equilibrium with the liquid hydrogen). Colder neutrons are preferred for experiments that study neutron decay properties because the neutron spends a longer time within the sensitive volume of the detector, thus increasing the probability of an observable decay.

**Fig. 1:**
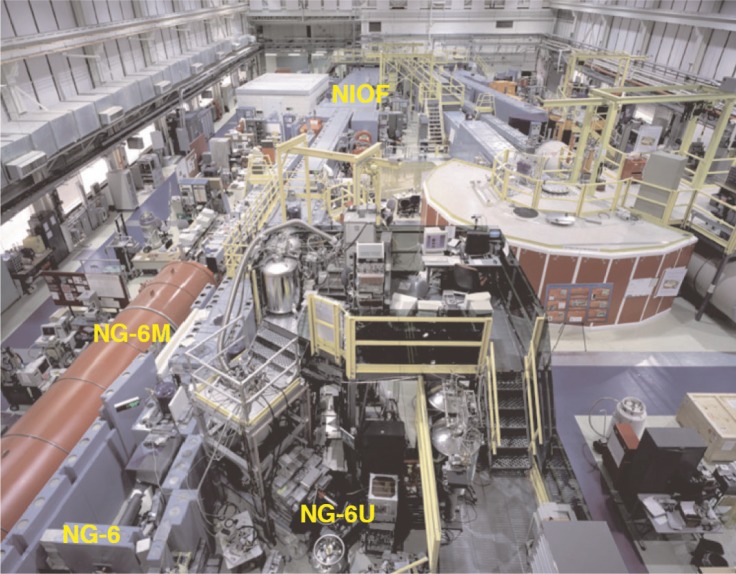
The neutron guide hall at the NCNR showing the beam lines and facilities for fundamental neutron studies.

After exiting the cold source, the neutrons enter evacuated rectangular guides (15 cm tall and 6 cm wide, coated with ^58^Ni) that transport the neutrons from the cold source to the experimental areas at neutron guide 7 (NG-7) or the end of neutron guide 6 (NG-6) on the NCNR neutron guide hall floor (see Fig. 1). ^58^Ni has an effective neutron potential of 335 neV such that neutrons with low enough momentum perpendicular to the surface will be totally reflected and thus can be transported to the far end of the guides (that are typically 20 m to 60 m in length) with minimal losses. For a fixed neutron wavelength, the surfaces of the guide reflect up to a critical angle of approximately 20 mrad nm^−1^.

At the NG-6 end station, the highly collimated cold neutron beam exits the guide through a thin Mg window and travels through 79 cm of air to an aperture that defines the beam diameter. The three monochromatic beams are obtained by the placement of monochromators in the beam path to Bragg reflect neutrons of a specific wavelength out of the beam. Upon exiting the NG-6 and NG-7 guide tubes, the neutrons are available for use at the experimental stations, as shown in Fig. 1. A description of those beams and some of their operating parameters are discussed below.

## 2. NG-6 Polychromatic Beam Line

**Fig. 2:**
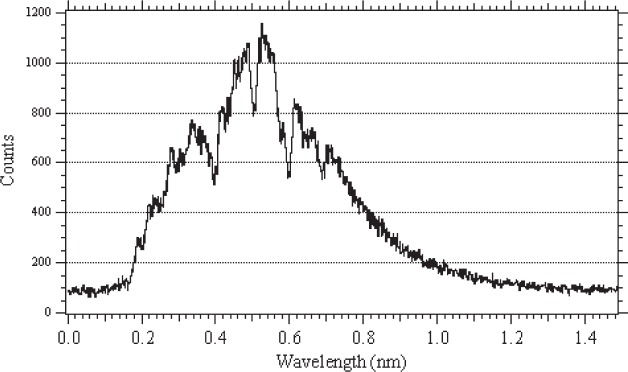
Wavelength spectrum of the NG-6 polychromatic beam. The dips in the spectrum correspond to Bragg-edges from materials upstream, typically aluminum and bismuth. The dip at 0.6 nm appears due to an upstream monochromator. The data were obtained with a 15 cm bismuth filter, polarizing supermirror, and neutron collimation in the beam.

The NG-6 beam is split into an upper and lower half for independent use by the end station and two monochromatic beams, respectively. Neutrons exiting from the upper 7.5 cm of the guide pass through a ^6^Li-loaded-glass collimator that defines a 6 cm diameter beam. The maximum fluence rate at a usable position on the beam line is approximately 2.3 × 10^9^ cm^−2^ s^−1^. The wavelength distribution of the beam is roughly Maxwellian with an average temperature of 40 K, and the peak wavelength is 0.5 nm. An example of the measured wavelength spectrum for a particular experimental configuration is shown in Fig. 2.

Just downstream of the defining collimator, there is a filter cryostat (77 K) that can hold up to 25 cm of filter material to produce more optimal conditions for a given experiment. Typical filter materials that have been used are bismuth, beryllium, and graphite. To minimize potential backgrounds to experiments, single-crystal bismuth filters may be placed in the beam to attenuate gamma rays and fast neutrons from upstream components in the guide. The blocks are cooled to reduce phonon scattering and thus reduce the loss of cold neutrons. Short wavelength neutrons can be filtered from the beam using Bragg scattering from polycrystalline materials, the most common being beryllium. Beryllium will Bragg reflect neutrons whose wavelengths are less than 0.395 nm out of the beam.

The facility supplies both polarized and unpolarized neutrons for experimenters. Two polarization technologies are available: supermirror and ^3^He transmission polarizers. Supermirror polarizers constructed at the Institut Laue-Langevin are used for both polarization and analysis. Polarization of 96 % is typical for the supermirror device. In this case, the polarized neutron beam becomes slightly more divergent and reflected by an angle of approximately 1° with respect of the primary beam. Polarized neutron fluence rates of 5 × 10^8^ cm^−2^ s^−1^ are routinely achieved for experiments.

In many applications, ^3^He transmission polarizers have several advantages over standard supermirror devices. They produce much less gamma background and do not add any additional divergence to the beam. They can also polarize large diameter beams and are effective over a broader wavelength spectrum than can be accommodated by supermirrors. The primary disadvantage of ^3^He polarizers has been the low figure-of-merit in comparison with supermirrors. Recent developments, however, have led to ^3^He polarization of 70 %, which yields a neutron polarization of 95 % with a neutron transmission of 21 %.

The experiments on NG-6 have all been studies of the weak interaction involving neutrons. The efforts include the measurement of the neutron lifetime, search for time-reversal violation, and studies of parity violation in the nucleon-nucleon system. The experiments are discussed in detail elsewhere and are treated only briefly here.

### 2.1 In-beam Neutron Lifetime Measurement

Two systematically distinct approaches to measuring the neutron lifetime were undertaken at NIST. The first used an in-beam technique with a proton trap and was recently completed. The second method is under development and involves three-dimensional magnetic confinement of ultracold neutrons. The in-beam method requires absolute counting of both neutrons and the decay protons. The cold neutron beam passes through a trap where decay protons are confined. The proton is held in a 4.6 T magnetic field that provides radial containment and electrodes at each end to provide axial confinement. Periodically, one of the end electrodes is lowered to ground potential to allow the accumulated trapped proton(s) to exit the trap and be counted by a proton detector. By knowing precisely the number of neutrons that decay in the trapping volume and the number that pass through, the neutron lifetime can be obtained.

The lifetime value obtained in this experiment is *τ*_n_ = (886.8 ± 3.4) s [2]. The dominant systematic uncertainties are related to neutron counting. The final uncertainty may be reduced by a factor of two as a result of continuing efforts to calibrate the neutron monitor by radiometric techniques.

### 2.2 Search for Time-Reversal Violation Invariance

The emiT collaboration was formed to improve the limit on the triple correlation *D*-coefficient. A beam of cold neutrons is polarized and collimated before it passes through a detection chamber with alternating electron and proton detectors. Electron-proton coincidence events are counted as a function of the neutron spin state to extract a measurement of *D*. The octagonal arrangement of the eight detector segments gives nearly full coverage of the 2π of azimuthal angle around the beam. In addition, the placement of the two types of detectors at relative angles of 135° produces a greater acceptance of proton-electron coincidences.

In 2000, the emiT collaboration published a result from the initial run of *D* = [−0.6 ± 1.2(stat) ± 0.5(sys)] × 10^−3^ [3]. The second run of the emiT experiment was recently completed with a greatly improved apparatus. The coincidence rate is approximately a factor of ten higher than in the first run, and the signal-to-background is 100:1. An analysis of the new data is underway, and the collaboration is optimistic that the current limit on *D* will be reduced significantly.

### 2.3 Parity-Violating Neutron Spin Rotation

Another effort seeks to measure the parity non-conserving rotation of the neutron spin as it passes through a liquid helium target. This angle of rotation is proportional to a linear combination of the weak meson exchange amplitudes *f* and *f*_π_, which is orthogonal to the combination recently constrained by the measurement of the ^133^Cs anapole moment. The initial run of this experiment was performed at NIST in 1996 and achieved a statistically limited null result of *ϕ*_PNC_ = (0.8 ± 1.4) × 10^−6^ rad/m [4]. Measurements showed that the systematic uncertainty on *ϕ*_PNC_ was quite small, about 2 × 10^−7^ rad/m. A substantial effort is underway to upgrade the apparatus in preparation for a second run with a goal on *ϕ*_PNC_ of 3 × 10^−7^ rad/m.

## 3. NG-6M 0.496 nm Monochromatic Beam Line

Two monochromators are positioned in the lower 7.5 cm of the polychromatic beam exiting the NG-6 guide. The upstream monochromator consists of a piece of pyrolytic graphic 5.1 cm by 5.1 cm by 0.2 cm thick with a mosaic of 0.8°. The crystal Bragg reflects a 0.496 nm beam at an angle of approximately 104° with respect to the primary beam.

Once the neutron beam reflects from the monochromator, it passes through 10 cm of 5 cooled beryllium to remove the *λ*/2 component of the beam. The neutron fluence rate available at a typical experimental position (approximately 2 m from the crystal) is 6.5 × 10^5^ cm^−2^ s^−1^. The 0.496 nm neutron beam is used for neutron radiometry experiments, ^3^He spin filter development, and neutron dosimetry work.

### 3.1 Absolute Neutron Fluence Measurements

The requirements of the NIST proton-trap lifetime experiment stimulated a wide-ranging effort to advance the state of the art of measurement of neutron fluence to the ±0.1 % level of accuracy. This challenge led to the development of new measurement techniques and the refinement of several established experimental procedures. A particularly significant development concerns the realization of two totally-absorbing or “black” neutron detectors with very well-known absolute efficiencies, a neutron radiometer [5] and the prompt gamma detector [6]. An essential feature of the fluence determination program is consistency among techniques; the cross calibration between the two detectors is necessary for reliability.

The neutron monitor employed in the neutron decay measurements is designed to measure the neutron density in the beam. Such a capture-fluence detector has an efficiency that is weighted by the reciprocal of the neutron velocity. The ^10^B and ^6^Li detectors used in the lifetime measurement are transmission devices that are nearly transparent to neutrons. In contrast, the “black” detectors have an efficiency which is essentially independent of incident neutron velocity. An intercalibration between the black and capture fluence detectors in a monochromatic beam is required.

While the motivation for the neutron fluence determination program has its origin in the neutron lifetime project, there is a number of other applications for the improved techniques. It would allow precise measurements of important neutron cross section standards at near-thermal energies, such as ^6^Li, ^10^B, and ^235^U. The technique of the black detectors essentially measures the product of the detector solid angle and the mass and cross section of the target material. This method can then be used to measure the cross section of any isotope that produces measurable reaction products in the capture-fluence detector.

### 3.2 Polarized ^3^He Program

Polarized neutrons are preferred or required for certain types of experiments in both fundamental physics and condensed matter studies. The ^3^He spin filter functions by the absorption of one spin state while passing the other state, so no deflection or divergence is introduced into the beam. Because of the simple inverse velocity relationship of the ^3^He-neutron cross section, it can serve well as a broadband neutron polarizer. There is an ongoing effort to develop practical neutron spin filters based on two different techniques for polarizing ^3^He: metastability-exchange optical pumping and spin exchange optical pumping. An overview of the two polarization techniques and a description of the neutron ^3^He program at NIST can be found in Refs. [7,8].

Polarized ^3^He spin filters are based on the spin dependence of the ^3^He-neutron capture cross section. Because the two neutrons in the ^4^He nucleus are spin paired, only neutrons of one spin state are absorbed by the ^3^He nucleus. For the ^3^He spin parallel to the neutron spin, the thermal capture cross section is essentially zero, whereas for the spins anti-parallel, the cross section is 10666 b. Using spin-exchange optical pumping, we have demonstrated 70 % to 75 % ^3^He polarization under the conditions that are relevant to neutron polarizers. One may also analyze the neutron polarization using ^3^He-based techniques.

## 4. NG-6U 0.89 nm Monochromatic Beam Line

Downstream of the 0.496 nm monochromator resides a 0.89 nm stage 2 potassium intercalated graphite monochromator [9]. The outgoing 0.89 nm neutron beam is 6 cm by 6 cm and is directed at an angle of 60° with respect to the polychromatic beam. The individual monochromator pieces have high stage purity and mosaics in the range of 1.1° to 2.1°. The reflectivity between 0.88 nm and 0.90 nm is (85.4 ± 0.5) %, yielding a capture fluence rate at 1 m from the crystal of 4.7 × 10^6^ cm^−2^ s^−1^.

**Fig. 3:**
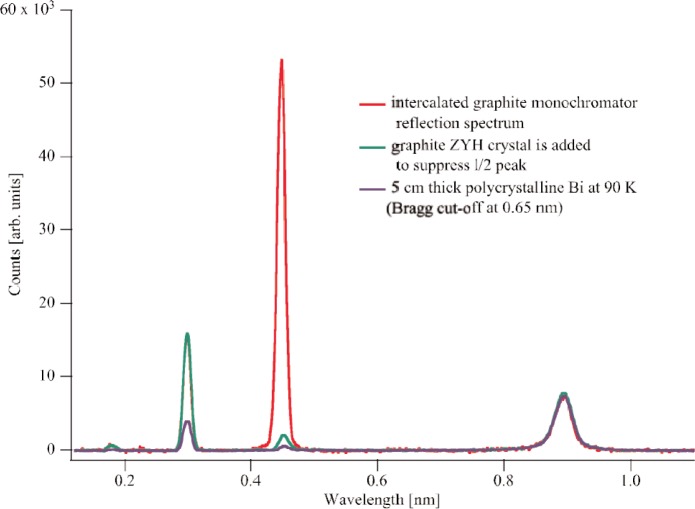
The wavelength spectrum of the 0.89 nm beam before and after filtering.

Two additional wavelengths are present, one at *λ*/2 = 0.45 nm and one at *λ*/3 = 0.30 nm. The *λ*/2 peak is removed by a pyrolytic graphite crystal with a 10° mosaic positioned to reflect out the 0.45 nm neutrons. The *λ*/3 peak is minimized using Bragg reflection from polycrystalline bismuth. A time-of-flight spectrum of the 0.89 nm neutron beam before and after wavelength filtering is shown in Fig. 3. The 0.89 nm beam is used primarily for ultracold neutron production for a new effort to measure the neutron lifetime, but some additional studies have been done for a proposed neutron electric dipole moment experiment [10].

### 4.1 Lifetime Measurement Using Magnetically Trapped Ultracold Neutrons

The second experiment under development for measuring the neutron lifetime uses magnetic trapping techniques. Ultracold neutrons are produced by inelastic scattering of cold (0.89 nm) neutrons in a reservoir of superfluid ^4^He (the “superthermal” process) [11]. These neutrons are confined by a three dimensional magnetic trap. As the trapped neutrons beta decay, the energetic electrons produced scintillations in the liquid He that are, in principle, detectable with nearly 100 % efficiency. The neutron lifetime can be directly determined by measuring the scintillation rate as a function of time. A proof-of-principle demonstration of this technique has been performed [12].

The apparatus is being upgraded so that more neutrons can be trapped. The main modification is a high-current quadrupole magnet that will be implemented to increase the trapping volume. The upgraded apparatus should produce an increase in the number of trapped neutrons by more than a factor of 20 without substantial increases in the background count rates. The goal of this experiment is to make a measurement of the neutron lifetime at the 10^−3^ level.

## 5. Neutron Interferometer and Optics Facility Monochromatic Beam Line

The Neutron Interferometer and Optics Facility is one of the world’s premier user facilities for neutron interferometry and related neutron optical measurements. A neutron interferometer splits and then recombines neutron waves. This gives the interferometer its unique ability to experimentally access the phase of neutron waves. Phase measurements are used to study the magnetic, nuclear, and structural properties of materials, as well fundamental questions in quantum physics. An overview of the facility can be found in Ref. [13].

**Fig. 4:**
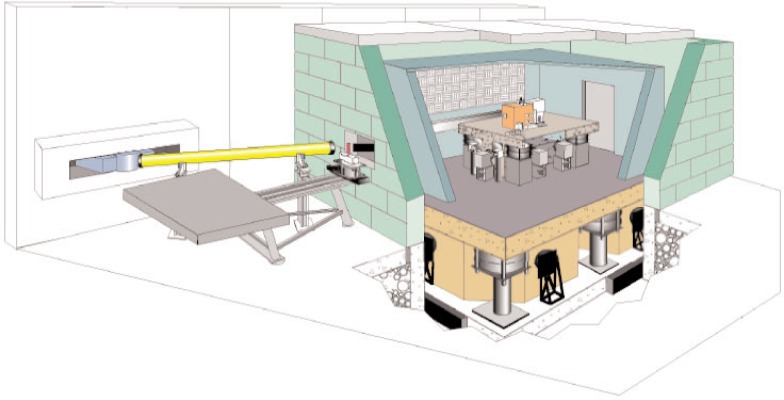
The neutron interferometer and optics facility.

Neutrons are extracted from the primary NG-7 beam using a dual-crystal parallel-tracking pyrolytic graphite monochromator system. Monochromatic beams (either unpolarized or polarized) with wavelengths from 0.2 nm to 0.48 nm are available. Neutrons are counted with integrating ^3^He detectors or by high-resolution position-sensitive detectors with a resolution better than 50 *µ*m. The sensitivity of the apparatus is greatly enhanced by state-of-the-art thermal, acoustic, and vibration isolation systems. To reduce vibration, the NIOF is built on its own foundation, separate from the rest of the building. The result is a neutron interferometry facility with exceptional phase stability (0.25°/d) and high contrast (90 %). The beam at the interferometer is 2 mm wide by 8 mm tall and has a fluence rate of 2 × 10^5^ cm^−2^ s^−1^. An illustration of the facility is shown in Fig. 4.

### 5.1 Precision Scattering Length Measurements

Neutron optical techniques provide a unique method for measuring the neutron bound coherent scattering lengths, *b*, of light elements. The technique of neutron matter wave interferometry is capable of measuring *b* for solids, liquids, or gases with a relative uncertainty ≤10^−4^.

Recent advances in effective field theories and Monte Carlo calculation techniques for two-body and three-body interactions among nucleons now make it possible to calculate neutron scattering lengths in low *A* nuclei from first principles. Results from measurements at NIOF of the n-p *b*_np_ = (−3.738 ± 0.002) fm and n-d *b*_nd_ = (6.665 ± 0.004) fm coherent scattering lengths [14] showed that essentially all existing calculations of the n-d coherent scattering length are in serious disagreement with experiment and that the accuracy of present measurements is sensitive to nuclear three-body forces. The precision of the recent measurement of the n-^3^He coherent scattering length *b*_n_3_He_ = (5.857 ± 0.007) fm is an improvement over previous measurements of almost an order of magnitude and is also sensitive to 3-nucleon forces in the *A* = 4 system. A separate measurement of the spin dependence of the n-^3^He scattering length can be used to determine precisely the amplitudes in both spin channels.

## 6. Conclusion

The Neutron Interactions and Dosimetry Group at NIST operates one end-guide position that is wholly dedicated to fundamental neutron physics research. This station presently consists of a primary high-flux polychromatic beam, a 0.49 nm monochromatic beam, and a 0.89 nm monochromatic beam for ultracold neutron production. The group also operates a state-of-the-art neutron interferometer and optics facility. The neutron beams are available to users, international and domestic, for experiments that fall in line with the general mission of the facility.

The common thread of the NIST experimental program is low energy investigations of the weak interaction and tests of the Standard Model through the study of neutron decay and neutron interactions. All the results have been a product of strong collaborations among university-based scientists, national laboratories, and NIST researchers. The strength of the collaborations (representing institutions in 11 states and 7 countries) and facility provides an excellent opportunity for postdocs to become independent researchers in neutron physics and to contribute to the training of graduate and undergraduate students.
